# X-Ray Therapy

**Published:** 1915-02

**Authors:** John M. Garratt

**Affiliations:** Buffalo, N. Y.


### BUFFALO MEDICAL JOURNAL

Yearly Volume 70. FEBRUARY, 1915. No. 7

### ORIGINAL ARTICLES

The right is reserved to decline papers not dealing with practical medical and surgical subjects, and such as might offend or fail to interest readers. Contributors are solely responsible for opinions, methods of expression and revision of proof.

### X-Ray Therapy.

JOHN M. GARRATT, M. D., Buffalo, N. Y.

Read before the Medical Union, November 24, 1914.

About ten days ago I was asked by one of your members to give an informal talk upon X-Ray therapy and to present some of the results obtained with the treatment. Obviously a paper prepared on such short notice must fail to properly present the subject, therefore I ask your kind indulgence for any short coming.

The development of X-Ray therapy dates from June, 1896, when Dr. Leopold Freund of Vienna applied the rays to the first patient, a girl with a large nevus covered with hair. The nevus was exposed for two hours daily for eleven days to the rays emanating from an ordinary Hittorf tube (capable of roentgenographing the adult hand with one minute's exposure). The hair was shed and a severe dermatitis resulted.

Today X-Ray therapy is a science: accurate measurement of the dose and filtering out the rays of short wave-lengths has given results hardly dreamed of a few years ago and all this without danger to the skin. By using the "cross-fire" technic deep seated growths can be given any desired dose. Surgery with a thorough after treatment with the X-Rays gives promise that the cure of cancer, in at least a large percentage of the cases, is near at hand.

The field of roentgenatherapy is a wide one and, therefore, only a few diseases can be presented this evening.

Roentgenotherapy, in my hands, has given prompt and satisfactory results in cases of

### Chronic Tubercular Cystitis.

Report of cases:

Male, age 31, with no tubercular family history. He complained of frequent micturition, during the day on an average of every hour and at night he had to get up four or five times. There was some difficulty in starting the flow of the urine and the voiding caused some smarting in the urethra, worst at the end of the act. The size of the stream varied but inclined to slight obstruction. Frequently there was a sudden stopping of the flow of urine before micturition was complete. Trouble was of six years standing. Examination of the urine showed an acid reaction with an average Sp. Gr. of 1.016 in five 24 hour samples and the sediment, which was considerable, was made up principally of pus-corpuscles loose and some in large loose clumps. Pus was drawn from the bladder through a sterile catheter and on staining tubercle bacillis were found in small numbers. No kidney elements—casts or renal epithelium were found.

Examination of the prostate through the rectum, found both lateral lobes moderately enlarged, soft and tender to the touch. The prostatic urethra was very sensitive on pressure. The seminal vesicles were nodular and tender.

The treatment—He was given urinary antiseptics and the bladder was irrigated. X-Ray applications were given every other day—raying alternately over the bladder and through the perineum. His progress—On the 10th day of treatment all discomfort had disappeared and micturition was normal as to frequency; on the 36th day the sudden stoppage of the flow had disappeared, the size of the stream had improved. Two years after stopping the treatment there had been no return of the symptoms.

Male, age 30; 20 lbs. under weight, which was normally 160 lbs. His trouble commenced five years before his history was taken. His physician had diagnosticated his case "stone in the bladder."

Symptoms: Constant pain in urethra at the meatus; when standing or moving about the pain was sharp and cutting; some relief was obtained the more he moved about, and on lying down. He had the sensation of foreign body in the bladder. Hematuria occasionally after violent exercise. No sudden stoppage of flow. Micturation about every hour during the day and 8 or 10 times during the night. Urine was scalding on voiding, very severe at end of act—so much so that he purposely did not empty his bladder.

Examination of prostate negative, seminal vesicles slightly indurated and tender.

24-hour samples of urine were examined on eight occasions. The average Sp. Gr. 1.022; reaction acid; of albumin there was a trace; sediment much—mostly pus, with a little normal blood, and a few pavement epithelium. Pus was stained of tubercle bacilli and they were found present in small numbers. No kidney elements were found. Roentgenographic examination of bladder for stone was negative.

Treatment as in the preceding case. In six weeks he had gained 17 lbs. Could hold his urine 2 hours during the day; average night micturation twice. Capacity of the bladder had increased from 2½ to 5½ oz. Sensation of foreign body had disappeared, also the discomfort in the urethra and on voiding urine. He returned to his home in Pennsylvania, with instructions to continue the bladder irrigations and urinary antiseptics. In six weeks he was in about the same condition as before treatment. He returned for further X-Ray treatment. In two months his weight was 165 lbs. and he was enjoying the best health he had experienced in five years.

### Lupus Vulgarus.

Report of cases:

Female, age 31. Heredity negative. Disease made its appearance following an attack of measles. During convalescence there appeared a redness on the side of nose at the junction with the lip; this was five years before the history was taken. It progressed as is usual in such cases. She had been under nearly constant medical treatment at home; in a Boston hospital for two weeks, and in Philadelphia she was under the care of one of America's most noted dermatologists for over a month. Healing would take place, only to relapse with renewed vigor.

The lesion extended from the upper lip to the inner canthus of the right eye. The soft parts of the nose, including both alae nasi were destroyed. The right cheek showed three active indolent ulcers, covered with reddish brown crusts. On the left cheek about ½ inch from the alae nasi was an ulcer ⅜ inch in diameter. Intervening the ulcers was scar tissue. There was no pain.

X-Rays were applied twice a week. At the 7th treatment the healing was gradual and uneventful, and complete at the 27th application.

Schoolgirl, age 18. Father died of phthisis. Previous history negative. Disease was of four years standing. First noted as a small pimple. It developed after the usual course, until, at the time of taking history, June 27, 1901, it covered two-thirds of the nose and upper lip. She was treated by her home physician and on two occasions it had apparently healed. It gave no pain, but there was a drawing sensation present. X-Ray treatments were given every other day. An ointment of sub-sulphate of mercury in lanolin was applied to soften the crusts. The crusts had disappeared after the third treatment. Healing was gradual and at the 17th treatment was nearly complete. There was an intermission in treatment for about a month. She had in all 58 X-Ray applications. Last treatment was November 2, 1901. There has been no recurrence.

Female, age 31. Married. Service of Dr. Francis Carr, who referred her to me for treatment. Family history.—One brother died of phthisis and her husband later developed consumption. No specific history. No severe sickness.

The lesion involved the greater part of both cheeks, forehead and chin; the soft part of the nose, left eye lid and the upper lip. The surfaces of the ulcers were covered with a brownish crust so that the base could not be seen; later the characteristic soft reddish brown papules and tubercles were defined.

This case was somewhat remarkable in that it had several earmarks of lues—it commenced inside of the nose, involving the bone, several small pieces being discharged and the roof of the mouth perforated making articulation most difficult. There was a serous, but not offensive discharge from the nostrils. The first treatment with the rays was given October 8, 1903. On October 22d the forehead and nose appeared to be healing rapidly and on November 21st all of the lesion was healed except at one corner of the lip. Treatment up to this time was given 3 times a week and was now reduced to once a week. January 4th, 1904, the upper lip started to break down and the rays were again given 3 times a week. On the 7th the lip began to slough rapidly. In addition to the rays she was now given potas. iodid. January 19th, sloughing was checked and by February 17th, the lip was nearly healed. May 20th the surface had all healed. Dr. Renner at this time examined the nose and reported a lesion in the left nostril ½ inch long. X-Rays were applied to it through a speculum and on July 6th it had healed. In all she had 68 rayings to the surface and 8 inside the nostril. There were no untoward effects. In December Dr. Monks of Boston made a new upper lip and lower eye lid. No recurrence.

### The Treatment of Recurrences and Metastases From Carcinoma of the Breast.

I cannot better describe the rational technic of the treatment of these conditions than to quote Dr. George E. Pfahler, of Philadelphia, in his paper read before the International Congress of Medicine in London, Eng., and published in the November issue of "Archives of the Roentgen Ray."

"The group of patients indicated by the above title is usually regarded as hopeless, and at the present time we must admit that no method of treatment is entirely satisfactory. Some remarkable results have been accomplished by the methods that I shall describe but these results are far from ideal, and in the end failure is the rule, and complete success the exception. Even with this dismal introduction, I am not without hope. More has been accomplished by the radio-therapeutic method than by any other treatment in this group of cases. The partial success obtained may point a way to a complete eradication and cure of the disease. In general the treatment consisted in the application of the roentgen rays as promptly and as vigorously as possible. The rays should be applied from as many directions as possible and carried to the limit of skin toleration, after filtration. To this has been added every measure that would tend to improve the patient's general health, so far as circumstances would permit. Combined with the above method I have added small doses of thyroid extract. . . . In the application of the rays for recurrent and metastases from carcinoma of the breast, much of the radiation reaches the thyroid gland and therefore, theoretically, would tend to diminish secretion. This fact, together with the fact that at the time of life when carcinoma is most common the thyroid secretion naturally tends to diminish, leads me to the belief that the administration of thyroid extract is distinctly indicated.''

During the past two years Dr. Pfahler has used the combined treatment in 150 cases with improvement in results to a considerable extent. He uses the Burrough's and Welcom's preparation, giving ½ grain three times a day, after meals. This is increased ½ grain every fourth day until 1½ grains are taken three times a day. Dr. Pfahler reports 15 cases in the advanced stages of the disease in which there has been no doubt as to a correct diagnosis—sections of the growth being made at the time of operation. For the most part the recurrences were extensive and inoperable. With the treatment the disease disappeared and the patient had, at least, a symptomatic cure. A few cases had the ray application without the thyroid extract and there had been no recurrence five years after treatment was discontinued. In one case the entire anterior chest wall and the right and left axilla were involved. In some of the cases there was involvement of the mediastinum and the results obtained are nothing short of remarkable. Results here as in other lesions are best obtained by attention to details and technic. In the first place a careful inspection and palpation of the entire chest and glandular regions is imperative and a roentgen examination of the chest and mediastinum —using a low vacuum tube and a plate rich in silver—is almost necessary and it gives a record from which future comparisons can be made. Having determined the extent of the disease, the rays should be directed from every angle—the anterior chest and axilla; posterior axillary and scapular and the supraclavicular regions. This will include the mediastinum in most every exposure. As a precaution against recurrence the rays should be applied immediately or as soon as possible after every operation of the breast in which malignancy is present or suspected. The spleen should be protected from the rays.

Report of cases:

Married woman, age 42. Referred to me for treatment by Dr. Chalmers. The disease was recurrent and situated in the right breast. Amputation was done on June 18th, 1914. Following the operation she received ten X-Ray treatments, the last one was applied July 22d.

At the time she presented herself to me there were two sinuses separated about two inches apart in the middle-third of the scar, these had been discharging for some time. One-half inch above the center of the scar was a nodular ridge 1½ inch long, which was quite painful. I gave her 15 X-Ray treatments between September 2d and November 22d. After the 7th both sinuses had healed and the ridge was reduced to ¾ inch in length and the nodules had entirely disappeared. The general health of the patient improved rapidly under the treatment and there is now no evidence of disease.

Married woman, age 58. Referred to me by Dr. Lothrop. Recurrent carcinoma. Amputation of the left breast by another surgeon 13 months before. A second operation was done by Dr. Lothrop on September 9th. She received the first X-Ray treatment on September 30th, at which time the dressing covered the wound—it was not disturbed, the margins of the skin were separated about four inches. She was weak and anemic. Healing was rapid under the treatment and she continued to gain in strength and color. On October 22d skin grafting was successfully done. October 28th she left for her home in a nearby town where X-Ray treatments are to be continued by her home physician.

### Uterine Myomata.

Dr. Arthur Holding of New York, in a recent article, reviews the literature of cases of myomata treated with the X-Rays, the total reported number, 794, of these 60 were still under treatment. The result of the treatment was known in 667 of which 376 or 56% were cured; 208 or 31% improved; 74 or 11% unimproved; 7 or 1% relapsed; 2 or .29% died and 52 or 7.8% developed an erythema. A very respectable showing, considering that the list includes good, bad and indifferent technic and diagnosis. From a study of the results accomplished in all varieties of tumors may be deduced indications and contraindications to the use of the treatment.

Indications: (a) All cases of myoma in older women in whom there is an advanced anemia.

(b) All elderly and young women in whom are found contraindications for an operation-marked organic heart disease, diabetes melitis, chronic kidney and lung diseases and goiter with cardiac symptoms.

The closer the approach to the menopause the more prompt and satisfactory is the result of the treatment. The intermural or interstitial varieties of tumor are most favorably influenced and in most cases completely disappear. In looking over the records we fail to find a single case in which malignant changes have occurred following X-Ray treatments. It is estimated that from one to two per cent. of myoma not so treated become malignant.

Contraindications: (1) Are the subserous, submucus and pedunculated varieties of tumor. Stein of New York reports the disappearance of a submucus tumor following X-Ray treatments.

(2) Tumors undergoing malignant changes or which are gangrenous should be promptly operated.

Choice of Technics:

**The Freiburg** or **Kronig-Gauss Technic** consists of from 300 to 500 or more X units administered in one or two days; this forms a series. After an interval of about 20 days another series is given. Usually five or six of such series are required to bring about a complete cure. The advantages claimed for this technic are: (a) results are obtained in the shortest possible time. (b) utilization of a greatly increased amount of X-Rays. (c) without danger to the skin. 10 X units or an erythema dose, well filtered, are administered over 21 skin areas, cross-firing, so as to direct the rays to the ovaries and tumor. The duration of the treatments for myoma would seem to be needlessly tedious to a sick woman, and it is not without danger, although brilliant results have been accomplished in the Freiburg clinic. In inoperable malignant conditions this technic has great possibilities.

**The Pfahler Technic** consists of but three abdominal areas—one over each ovary, and one directed to the tumor—and a fourth to the perineum; the four areas may be treated in one or two days to complete a series. There is less danger of overlapping of the lines of radiation. Results are obtained in four or five months. This is the technic which I prefer, and was used in the treatment of the cases, the reports of which follow.

The favorable time to apply the treatment is just after menstruation, and ten days before. If the bleeding has been nearly constant, treatment may be applied at any time.

Report of cases:

Female, age 44, single. Referred by Dr. Hoag. Tumor reached one inch above the umbilicus after three years growth. Had nearly constant hemorrhages for the past three months—losing large quantities of blood; very anaemic, hemoglobin 50%. Also under treatment for stomach trouble.

Progress. First series given February 14th, 1914, after which all excessive hemorrhage stopped; after the 2nd menstruation was normal, lasting six days. Improvement in general health was gradual, with reduction of the size of the tumor. After the 12th series, given July 8th, I could not feel the tumor through the abdominal wall; the same day she was examined by Dr. Hoag, who reported the entire disappearance of the growth. Hemoglobin 90%, and the patient was apparently restored to perfect health.

Female, age 42, Married. Referred by Dr. Hoag. This tumor, which reached to one-half inch of the umbilicus, was of 4 years growth. About a year ago she received between 25 and 30 uterine electrical treatments with no result except to cause pain. Menstruation usually lasted 10 to 14 days and there was a profuse flow the 2d and 3d days. There was a continuous loss of blood from August 29th to October 12th, the day she received the 1st series of the treatment, when it was checked. Two weeks before she passed five large clots having the appearance of "black paint." Patient was weak and wanted to sleep all of the time. She has had five series of treatments. After the second menstrual flow was more profuse than usual but stopped the sixth day. At the last treatment the tumor had decreased so that the upper border was 3 inches below the umbilicus. She was in better strength and color.

The advantages of this method of treatment: It is painless, and if pain is a symptom and if due to hypersensitive ovaries the relief is prompt; there is no shock; **internal secretion of the ovaries is preserved**; occupation and earning power of employed women is not interfered with; menopause is brought on gradually. Menstruation is re-established in some cases and patients later may become pregnant and give birth to healthy children. One case was reported in which the patient became pregnant during treatment and the foetus unsuspectedly received three series of raying, the child showed no signs of injury and was apparently healthy and 4 months old when the case was reported.

---

**Movement of Enemata.** Drummond, Brit. Med. Jour., Jan. 31, 1914, investigating faecal fisulae and using X-ray methods finds that 1½-2 pint enemata, injected slowly into the rectum, normally reach the caecum in ten minutes but do not pass the ileo-caecal valve, if competent. For some time after ileo-colostomy, the ileum being implanted low in the pelvic colon, enemata do not pass into the ileum but after a year or more has elapsed, they do reach the lower part of the ileum, which seems to assume the functions of the upper colon and caecum.

